# Supplementary material for: Activation of CD8+ T Cell Responses after Melanoma Antigen Targeting to CD169+ Antigen Presenting Cells in Mice and Humans
Source: Cancers (Basel). 2019 Feb 5;11(2):183. doi: 10.3390/cancers11020183 (PMC6406251; doi:10.3390/cancers11020183)
Supplement: Supplementary file 1 [file cancers-11-00183-s001.pdf]

cancers

MDPI

Supplementary Materials: Activation of CD8+ T Cell Responses after Melanoma Antigen Targeting to CD169+ Antigen Presenting Cells in Mice and Humans

Dieke van Dinther, Miguel Lopez Venegas, Henrike Veninga, Katarzyna Olesek, Leoni Hoogterp, Mirjam Revet, Martino Ambrosini, Hakan Kalay, Johannes Stöckl, Yvette van Kooyk and Joke M. M. den Haan

Table S1. Number of biotinylated peptides per indicated Ab:Ag conjugate.

Antibody:Antigen Conjugate	#Peptides
R7D4:hgp100	2.4
NLDC-145:hgp100	3.2
MOMA-1:hgp100	1.8
R7D4:Trp2	1.3
NLDC-145:Trp2	3.8
MOMA-1:Trp2	2.4
R7D4:Trp1	2.1
NLDC-145:Trp1	1.8
MOMA-1:Trp1	1.3
R7D4:MART1	<1
NLDC-145:MART1	<1
MOMA-1:MART1	<1
10E2:gp100	1.3
7-239:gp100	1.7
AZND1:gp100	1.5

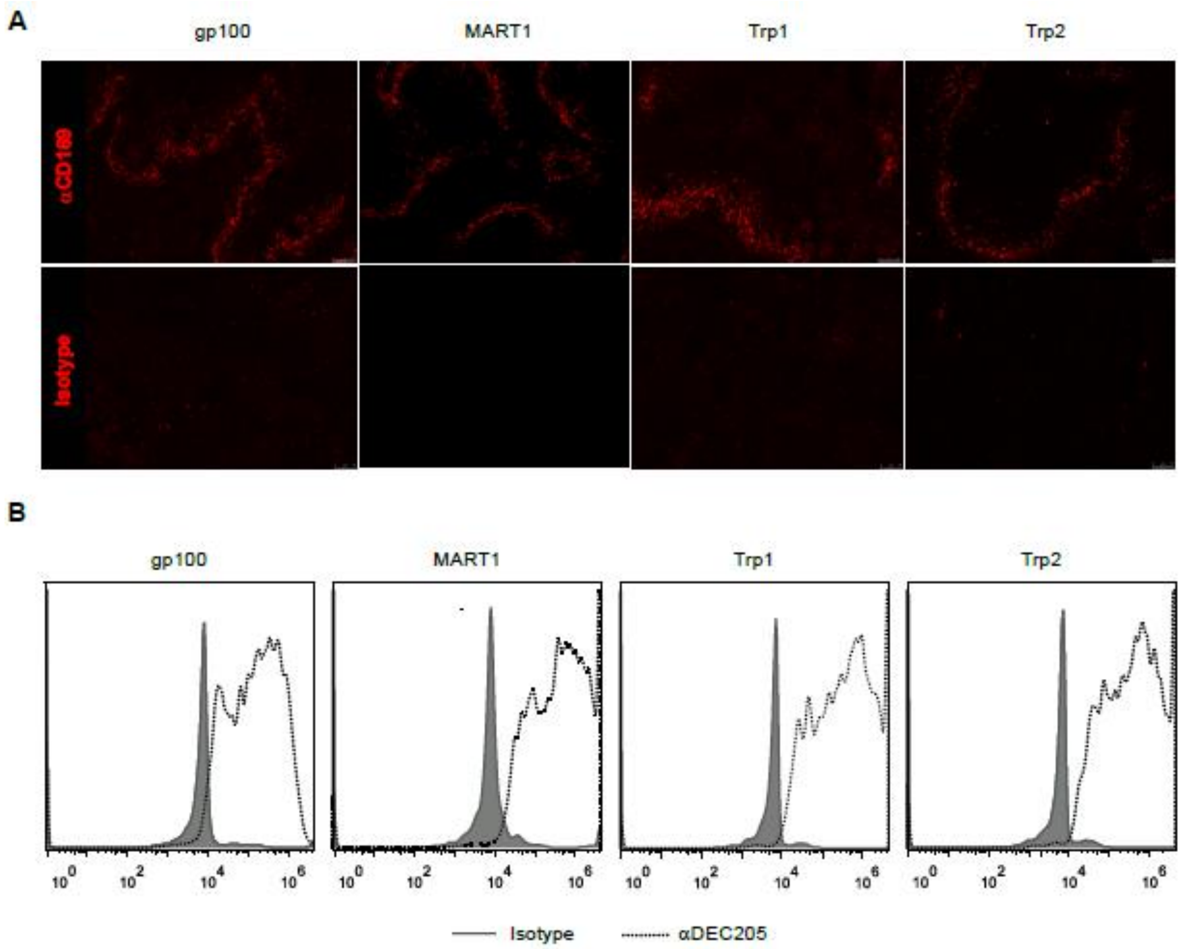

Figure S1. Chemical conjugation of melanoma peptides to Ab does not alter binding of Ab. (**A**) section staining on mouse spleen with Ab:Ag conjugates specific for CD169 (upper panels), or isotype control (lower panel) with indicated peptides. (**B**) staining on CHO cells expressing DEC205 with Ab:Ag conjugates specific for DEC205 with indicated peptides.

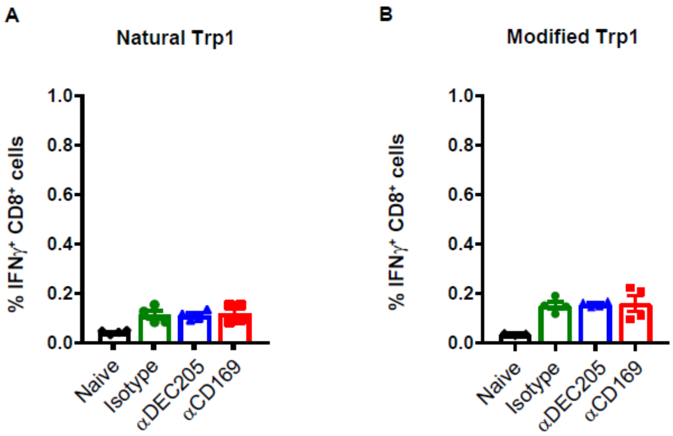

Figure S2. T cell responses in mice after targeting Trp1 to CD169. Percentage of IFNγ producing CD8+ T cells after 5 h in vitro restimulation with natural Trp1 peptide (**A**) or modified Trp1 peptide (**B**) 7 days after immunization with Trp1:Ab conjugates. Intravenous immunization with 1 μg modified Trp1:Ag conjugates in the presence of 25 μg anti-CD40 Ab and 25 μg Poly(I:C).

© 2019 by the authors. Licensee MDPI, Basel, Switzerland. This article is an open access article distributed under the terms and conditions of the Creative Commons Attribution (CC BY) license (http://creativecommons.org/licenses/by/4.0/).
